# Supplementary material for: The place-cell representation of volumetric space in rats
Source: Nat Commun. 2020 Feb 7;11:789. doi: 10.1038/s41467-020-14611-7 (PMC7005894; doi:10.1038/s41467-020-14611-7)
Supplement: Supplementary file 3 — Reporting Summary [file 41467_2020_14611_MOESM3_ESM.pdf]

nature research

Corresponding author(s): Roddy M. Grieves
Kate J. Jeffery

Last updated by author(s): Jan 10, 2020

Reporting Summary

Nature Research wishes to improve the reproducibility of the work that we publish. This form provides structure for consistency and transparency in reporting. For further information on Nature Research policies, see Authors & Referees and the Editorial Policy Checklist .

Statistics

For all statistical analyses, confirm that the following items are present in the figure legend, table legend, main text, or Methods section.

n/a	Confirmed	
☐	☒	The exact sample size (*n*) for each experimental group/condition, given as a discrete number and unit of measurement
☐	☒	A statement on whether measurements were taken from distinct samples or whether the same sample was measured repeatedly
☐	☒	The statistical test(s) used AND whether they are one- or two-sided<br>*Only common tests should be described solely by name; describe more complex techniques in the Methods section.*
☐	☒	A description of all covariates tested
☐	☒	A description of any assumptions or corrections, such as tests of normality and adjustment for multiple comparisons
☐	☒	A full description of the statistical parameters including central tendency (e.g. means) or other basic estimates (e.g. regression coefficient) AND variation (e.g. standard deviation) or associated estimates of uncertainty (e.g. confidence intervals)
☐	☒	For null hypothesis testing, the test statistic (e.g. *F*, *t*, *r*) with confidence intervals, effect sizes, degrees of freedom and *P* value noted<br>*Give P values as exact values whenever suitable.*
☒	☐	For Bayesian analysis, information on the choice of priors and Markov chain Monte Carlo settings
☒	☐	For hierarchical and complex designs, identification of the appropriate level for tests and full reporting of outcomes
☐	☒	Estimates of effect sizes (e.g. Cohen's *d*, Pearson's *r*), indicating how they were calculated

Our web collection on statistics for biologists contains articles on many of the points above.

Software and code

Policy information about availability of computer code

Data collection	Descriptions of software are given in text. Mainly; dacqUSB (Axona), dacqTrack (Axona)
Data analysis	Descriptions of software are given in text. Mainly; Tint (v.4.4.12, Axona), Matlab (2018a, The Mathoworks), SPSS (v25, IBM)<br>Matlab code for analysis can be downloaded from: DOI: 10.5522/04/9975398

For manuscripts utilizing custom algorithms or software that are central to the research but not yet described in published literature, software must be made available to editors/reviewers. We strongly encourage code deposition in a community repository (e.g. GitHub). See the Nature Research guidelines for submitting code & software for further information.

Data

Policy information about availability of data

All manuscripts must include a data availability statement. This statement should provide the following information, where applicable:

- Accession codes, unique identifiers, or web links for publicly available datasets
- A list of figures that have associated raw data
- A description of any restrictions on data availability

The full data set, in its analyzed form, can be downloaded from DOI: 10.5522/04/9926477; this includes all of the data reported or presented in the main text or supplementary files. The full raw data set is available from the authors on request.

Field-specific reporting

Please select the one below that is the best fit for your research. If you are not sure, read the appropriate sections before making your selection.

Life sciences study design

All studies must disclose on these points even when the disclosure is negative.

Sample size	Groups of 4 animals or more are typical in hippocampal research where we would also aim for 200 or more cells per treatment. We collected data from 9 animals for the main experiment and >400 place cells and 4 animals in the control condition with >250 place cells. No a priori power analysis was undertaken.
Data exclusions	No animals or place cells were excluded. Data clusters that were not classified as place cells were excluded.
Replication	Replication was not undertaken in this study although our effects are present across animals and sessions.
Randomization	Animals were randomly assigned to the aligned and tilted lattice groups.
Blinding	The experimenters also analyzed the data and were not blinded.

Reporting for specific materials, systems and methods

We require information from authors about some types of materials, experimental systems and methods used in many studies. Here, indicate whether each material, system or method listed is relevant to your study. If you are not sure if a list item applies to your research, read the appropriate section before selecting a response.

Materials & experimental systems

n/a	Involved in the study
☒	☐ Antibodies
☒	☐ Eukaryotic cell lines
☒	☐ Palaeontology
☐	☒ Animals and other organisms
☒	☐ Human research participants
☒	☐ Clinical data

Methods

n/a	Involved in the study
☒	☐ ChIP-seq
☒	☐ Flow cytometry
☒	☐ MRI-based neuroimaging

Animals and other organisms

Policy information about studies involving animals; ARRIVE guidelines recommended for reporting animal research

Laboratory animals	Lister hooded rats, male, 250-400g in weight, age unknown
Wild animals	The study did not involve wild animals
Field-collected samples	The study did not involve samples taken from the field/wild.
Ethics oversight	Animal care technicians, Named Veterinary Surgeons and Named Animal Care and Welfare Officers provided ethical and care guidance. This experiment complied with the national [Animals (Scientific Procedures) Act, 1986, United Kingdom] and international [European Communities Council Directive of November 24, 1986 (86/609/EEC)] legislation governing the maintenance of laboratory animals and their use in scientific experiments.

Note that full information on the approval of the study protocol must also be provided in the manuscript.
