# Supplementary material for: The place-cell representation of volumetric space in rats
Source: Nat Commun. 2020 Feb 7;11:789. doi: 10.1038/s41467-020-14611-7 (PMC7005894; doi:10.1038/s41467-020-14611-7)
Supplement: Supplementary file 4 — Description of Additional Supplementary Files [file 41467_2020_14611_MOESM4_ESM.pdf]

Description of Additional Supplementary Files

File Name: Supplementary Movie 1
Description: An example trajectory from a square arena, aligned lattice and tilted lattice session respectively. Alongside the sped up play through of the trajectory (top left) is included the instantaneous heading direction of the animal (top right) the spherical heatmap of the current heading direction (bottom left) and spherical heatmap of all trajectory headings so far (bottom right).

File Name: Supplementary Movie 2
Description: Example footage recorded from different cameras of rats performing the task; exploring the open arena or aligned lattice maze.

File Name: Supplementary Movie 3
Description: Example place cells recorded in the aligned lattice shown in three dimensional rotating plots where it is easier to appreciate their 3D firing structure.

File Name: Supplementary Movie 4
Description: Example place cells recorded in the tilted lattice shown in three dimensional rotating plots where it is easier to appreciate their 3D firing structure.
